# Zika and women's sexual and reproductive health: Critical first steps to understand the role of gender in the Colombian epidemic

**DOI:** 10.1002/ijgo.13043

**Published:** 2020-01-23

**Authors:** Luz J. Forero‐Martínez, Rocío Murad, Mariana Calderón‐Jaramillo, Juan C. Rivillas‐García

**Affiliations:** ^1^ Asociación Profamilia Bogotá D.C Colombia

**Keywords:** Climate change, Colombia, Gender, Sexual and reproductive health, Sustainable Development Goals, Zika virus

## Abstract

**Objectives:**

To describe the mechanisms of implementation of Zika virus diagnosis, prevention, and management guidelines in Colombia, and to characterize their influence on efforts to defend sexual and reproductive rights.

**Methods:**

A qualitative study performed between February and April 2018 in three municipalities in Colombia. We conducted 30 semistructured interviews and five focus groups with key informants who played a role during the epidemic. These included decision‐makers, program coordinators, healthcare providers, pregnant women diagnosed with Zika virus, and members of affected communities.

**Results:**

We identified barriers to and facilitators for the implementation of the national Zika virus response plan. Barriers included a lack of coordination between vector control efforts and in the realms of sexual and reproductive rights. Facilitators included healthcare providers’ response to the epidemic, the development of technical skills, and the establishment of coordination and referral networks across different institutions.

**Conclusion:**

A multidimensional approach that considers healthcare services, gender issues, and the environment is crucial. We highlight the epidemic's effects on women's sexual and reproductive rights, mainly related to inequalities in sexual and reproductive health such as the increased risk of sexually transmitted infections experienced by the poorest and most vulnerable women.

## Abstract 2

The Zika virus epidemic has different effects on women's and men's reproductive health. This calls for a multidimensional approach to the epidemic.

## 1 INTRODUCTION

The global agenda of the Sustainable Development Goals (SDGs) for 2030 emphasizes the importance of understanding the interactions between gender, sexual and reproductive health (SRH), and efforts to mitigate climate change.^1^ Vulnerability to the forces of climate change and to emerging epidemics is not determined exclusively by biology. Differences in roles and responsibilities expose women and girls to additional risks.^2^ In addition to gender, other axes of power and inequalities such as race, socioeconomic status, occupation, age, disability, and sexual preference all influence people's vulnerability.^3^ This makes the intersectional analysis of global health problems, including SRH, extremely relevant.^4^

In poor communities in northeast Brazil, an increase in the number of infants born with microcephaly during a Zika virus epidemic led to the discovery that Zika virus infection during pregnancy was the cause. Currently, Zika virus exists in 69 countries, and birth defects linked to infection during pregnancy have been described in 29 countries.^5^ The sexual transmission of Zika virus has been documented,^6^, ^7^, ^8^ but there are additional implications for women's SRH that have not been reported and which are related to inequalities in SRH. These include the increased risk of sexually transmitted infections; barriers to accessing quality primary healthcare; lack of adherence to programs and protocols; and stigma and discrimination experienced by the poorest and most vulnerable women. Climate change also impacts the health of women in low‐ and middle‐income countries.^2^, ^9^

In Colombia, between 2015 and 2017, vector‐borne diseases like malaria, dengue, chikungunya, and Zika virus affected more women than men.^10^ For Zika virus, since the beginning of the epidemic phase, 66.4% of suspected and confirmed cases were in women.^11^ In total, there were 6363 confirmed cases and 13 383 suspected cases among pregnant women who reported some symptoms compatible with Zika virus infection.^11^

Zika virus has profound consequences for women's SRH^12^, ^13^ because it is sexually transmitted^6^, ^7^, ^8^ and carries high risks for pregnant women^14^, ^15^ through mother‐to‐child transmission, which can cause microcephaly and other central nervous system anomalies.^16^, ^17^, ^18^ In addition, the emergence of Zika virus is related to environmental causes and climate change, which in turn influence the epidemic and women's SRH. For these reasons, a multidimensional approach to the epidemic is warranted.

In this context, a plan to address Zika virus should go beyond classic health‐related actions or vector control strategies (such as improving living conditions and water sources) and should incorporate actions that address sexual and reproductive rights (SRR). Addressing the epidemic should include recognition that Zika virus affects women and men differently, that the virus is sexually transmitted, and that early diagnosis of symptomatic pregnant women is key, as is access to contraception and safe abortion.

In 2016 the Colombian government prioritized Zika virus in the political agenda and started the implementation of the Zika Virus Fever Response Plan.^19^ The focus of the plan was to strengthen public health surveillance systems for vector‐transmitted diseases and to increase health education, prevention, and vector control efforts. The program also included funding for supplies such as insecticides, larvicides, and mosquito nets. It also addressed contingencies derived from climate change and encouraged research and monitoring of pregnant women and children's health.

The present study puts forth a multidimensional approach to Zika virus as a first step to understanding the epidemic's impact on SRH issues. The objective was to describe the mechanisms of implementation of the national guidelines to detect, prevent, and treat Zika virus; then describe their impact on SRR in Colombia. The focus was on three municipalities that were affected differently by the epidemic: Barranquilla, Cúcuta, and San Andrés.

## 2 MATERIALS AND METHODS

This qualitative study was conducted in Colombia between February 21 and April 10, 2018. The study was approved by the Pan‐American Health Organization Ethical Review Committee (PAHOERC) and the Ethical Committee of Profamilia. All participants signed an informed consent form. We conducted semistructured interviews and focus groups with key informants who belonged to one of the following groups: (1) national government; (2) local/regional government; (3) healthcare providers; (4) community members; and (5) women diagnosed with Zika virus. We used study instruments to identify facilitators and barriers in the implementation of the National Zika Virus Fever Response Plan,^10^ as well as any actions taken to guarantee the SRR of pregnant women affected by or at high risk of Zika virus.

We conducted a total of 25 interviews (17 women and eight men) with decision‐makers, those responsible for the development and implementation of the Response Plan, and representatives of local health departments, insurers, and healthcare providers. Respondents also included coordinators of vector‐borne disease programs and other public health surveillance, education, and prevention specialists. We also interviewed five pregnant women with a confirmed Zika virus diagnosis and conducted five focus groups with community leaders and community members.

We recruited respondents according to location and logistical capabilities and continued recruiting until we reached thematic saturation. In San Andrés the local health department took the lead in recruitment, while in the other two municipalities Profamilia oversaw recruitment. All interviews were transcribed and then analyzed using ATLAS.ti software 7.5.4 (ATLAS.ti Scientific Software Development GmbH, Berlin, Germany).

## 3 RESULTS

We identified facilitating factors that spurred the implementation of actions to address the Zika virus epidemic. We also identified barriers and aspects to improve in the implementation process.

### 3.1 Facilitating factors

The following factors spurred and facilitated the implementation of actions to address the Zika virus epidemic: (1) the healthcare sector's response; (2) development of technical capabilities; and (3) communication among different institutions.

#### 3.1.1 Healthcare sector response

In all study locations, healthcare providers and community members all agreed that the needs of pregnant women needed to be prioritized because they represent the highest risk group. Some respondents highlighted that healthcare providers emphasized SRR by conducting educational activities with affected communities. These included information about delaying pregnancy, using barrier methods of contraception, and abortion. Educational campaigns were also conducted outside of healthcare centers, and healthcare personnel received training on Zika virus. These activities reduced barriers to care and enhanced the detection of cases among pregnant women as well as their follow‐up care.

#### 3.1.2 Development of technical teams

National and local health authorities established agreements and coordinated efforts to conduct trainings on Zika virus and develop programs to care for these individuals. As a result, technical skills and capabilities were enhanced at a national level. The national Ministry of Health also collaborated with the Centers for Disease Control and Prevention (CDC) in the USA on a program to identify pregnant women at risk and detect the consequences of infection (microcephaly or other nervous system defects) through early diagnosis. Epidemiologic data were used to establish clinical protocols to care for pregnant women, which included early diagnosis and treatment of the disease.

Teams of technical experts also improved their skillset by working at the local and regional level. After the first cases were reported, efforts were made to characterize the virus and devise epidemiologic surveillance and clinical practice protocols. However, obstacles were encountered with sending biologic specimens to the National Institute of Health in Bogotá, and long wait times were noted before a confirmatory result was available. This impacted the clinical follow‐up of affected patients.

#### 3.1.3 Interinstitutional cooperation

Public and private healthcare providers and research institutions coordinated efforts and shared objectives, which helped strengthen the surveillance capacity of the public health system as well as the capacity to diagnose and treat cases at the health system level. This also allowed members of varying institutions to access the trainings described above. At the national and regional level, as well as among healthcare providers, there was consensus that the Ministry of Health and the National Institute of Health had been key in determining the successful implementation of national guidelines.

### 3.2 Barriers to implementing the response plan

The following paragraphs describe the three barriers that emerged, as a critique to the implementation of the National Zika Virus Response plan: (1) focus on sexual and reproductive health; (2) focus on regional initiatives; and (3) attitudes and knowledge at the community level.

#### 3.2.1 Focus on human rights and sexual and reproductive rights in the National Response Plan

The profound implications of Zika virus on SRR were not completely recognized and integrated into the Response Plan; for example, recognition that Zika virus is a sexually transmitted infection and that access to health services is key for the affected population. Zika virus outbreak prevention and management efforts focused on vector control rather than on SRH consequences.

The focus on SRH was promoted at the national level, but these efforts were not sufficiently integrated with the National Policy on Sexuality, Sexual and Reproductive Rights.^20^ One of the barriers was religious and cultural beliefs, which stigmatized abortion as an option for pregnant women infected with Zika virus. Some healthcare providers admitted that they did not provide patients with comprehensive information, and that their counseling focused on the newborn's microcephaly risk instead.

#### 3.2.2 Local/regional efforts focused primarily on vector control

Training and preparation efforts were primarily oriented toward developing competencies directly related to Zika virus, rather than toward the epidemic's impact on women's SRR. All of the interviewees discussed Zika virus vector control programs, including cleanup of mosquito breeding grounds and management of water and sewage systems. Community education programs encouraged people to wear covering clothing and mosquito repellent to prevent bites. Public information campaigns included information about how to reduce the risk of infection, how to identify the symptoms, and how to access healthcare services. These campaigns targeted people who lived in or transited through areas at high risk of transmission.

#### 3.2.3 Attitudes, behaviors, and knowledge at the community level

In terms of attitudes, what stood out was the use of knowledge to implement preventative actions against the spread of the vector, as well as the attention to the disease at the individual, family, and community level. However, lack of information and barriers to accessing services persisted, and some infected individuals who did not access healthcare services decided to self‐medicate with “home remedies.”

Pregnant women with suspected or confirmed infection expressed the need to reinforce communication and prevention campaigns and to focus on their needs as women. We also found that the modes of transmission of the virus were not well explained to the public, and the recommendation to use barrier methods of contraception was not strong enough. In summary, the response to Zika virus at the community level and in terms of personal behavior change was inadequate.

## 4 DISCUSSION

Our study identified facilitating factors for and barriers to the implementation of the National Response Plan to the Zika virus epidemic in Colombia. The most significant lesson learned was that a multidimensional approach to Zika virus is necessary to address the interaction of the epidemic with gender roles, sexual and reproductive health and rights, and the environment.

In addition to providing health care, providers engaged in health education and prevention campaigns. They achieved some success in terms of aligning public health efforts to address Zika virus with efforts to defend sexual and reproductive health and rights and to improve the health system's resilience during the epidemic. However, the National Zika Virus Response Plan was not aligned with the National Policy on Sexual and Reproductive Rights^20^ and with the Ten‐Year Public Health Plan 2012–2021.^21^ This resulted in an increase in vulnerability and worse health outcomes for pregnant women and subsequently their newborns.

In the communities, knowledge about SRR in the context of Zika virus was reduced to abortion. However, access was limited because the issue is stigmatized, and women were not well informed about their right to access safe abortion. SRH was not understood as a broad concept, which resulted in the loss of opportunities to provide care and promote contraceptive counseling among women in affected areas.

Our findings are aligned with those described by Vilela Borges et al.,^13^ who evaluated knowledge and attitudes about pregnancy and contraception in the context of Zika virus in Brazil. In that study, health officials informed the public about Zika virus transmission and its health consequences, and providers knew about the congenital syndrome, but knowledge about sexual transmission was low.

At the community level, the absence of effective, gender‐focused messages to prevent Zika virus reinforced stereotypes and inequalities. Healthcare services need a more strategic focus to reach communities early, to broaden their offer of counseling and contraceptive services, and to eliminate barriers to accessing abortion in the context of an epidemic. Health programs that address Zika virus and other epidemics must recognize that affected women and men have different needs and that there can be a significant impact on women's SRH. Furthermore, it is crucial that knowledge and research findings reach affected communities.

## AUTHOR CONTRIBUTIONS

RM and LF‐M conceptualized the research project. RM directed the fieldwork and data collection. MC‐J analyzed the data. JR‐G edited the original manuscript. All authors contributed to the manuscript and approved the final version.

## CONFLICTS OF INTEREST

The authors have no conflicts of interest.
